# Development of a multiplex serological assay reveals a worldwide distribution of murine astrovirus infections in laboratory mice

**DOI:** 10.1371/journal.pone.0187174

**Published:** 2017-10-31

**Authors:** Katja Schmidt, Julia Butt, Petra Mauter, Klaus Vogel, Andrea Erles-Kemna, Michael Pawlita, Werner Nicklas

**Affiliations:** 1 Microbiological Diagnostics, Center for Preclinical Research, German Cancer Research Center, Heidelberg, Germany; 2 Division of Molecular Diagnostics of Oncogenic Infections, German Cancer Research Center, Heidelberg, Germany; California State University Fresno, UNITED STATES

## Abstract

Laboratory mice play a tremendous role in biomedical research in studies on immunology, infection, cancer and therapy. In the course of standardization of mice used in animal experiments, health monitoring constitutes an important instrument towards microbiological standardization. Infections with murine astroviruses (MuAstV) were only recently discovered and are, therefore, still relatively unknown in laboratory animal science. In rodent health monitoring viral infections within a population are commonly assessed in terms of specific antibodies by serological testing, as active infection and excretion of virus is often temporary and can easily be missed. So far only ongoing infections with astroviruses can be detected by PCR. The objective of this work was the development of a sensitive and specific MuAstV multiplex serological assay with a high-throughput capability to be used in routine testing of laboratory mice. Four different MuAstV proteins were recombinantly expressed and used as antigens. The best reacting antigen, the capsid spike protein VP27, was selected and tested with a panel of 400 sera of mice from units with a known MuAstV status. Assay sensitivity and specificity resulted in 98.5% and 100%, respectively, compared to RT-PCR results. Eventually this assay was used to test 5529 serum samples in total, during routine diagnostics at the German Cancer Research Center (DKFZ) in Heidelberg between 2015 and 2017. High sero-prevalence rates of up to 98% were detected in units with open cages indicating that the virus is highly infectious and circulates within these populations virtually infecting all animals regardless of the mouse strain. In addition, data collected from 312 mice purchased from commercial breeders and from 661 mice from 58 research institutes in 15 countries worldwide allowed the conclusion that MuAstV is widespread in contemporary laboratory mouse populations.

## Introduction

Infections with astroviruses, non-enveloped, single-stranded RNA viruses of the family *Astroviridae*, are a leading cause of gastroenteritis in humans. Astroviruses were first described in 1975 following an outbreak of diarrhea in children [1, 2]. Since their first discovery, astroviruses were isolated from a wide range of mammalian and avian species including mink, chicken, cat, dog, deer, duck, turkey, sheep, cattle and bat [3]. Based on their host tropism and genetic relatedness astroviruses are grouped in two genera, *Mamastrovirus* and *Avastrovirus*, infecting mammals and birds, respectively [4]. Rodent-associated astrovirus (murine astrovirus, MuAstV) was first described by Kjeldsberg and Hem in 1985 [5], but only recently raised awareness in laboratory animal science when detected in fecal samples of wild and laboratory mice by use of metagenomic analysis and PCR [6, 7]. Molecular biological analyses suggest that murine astroviruses are widespread in research facilities in Japan and the USA and that, in particular, immunodeficient mice are infected [8]. As a result of the lack of serological detection methods so far only active infections with virus excretion can be detected by PCR and thus prevalence and distribution within laboratory mice populations are unclear.

Regardless of a possible interest in an animal model for human medicine research, this new virus may also play a role in health monitoring of laboratory mice in near future. Health surveillance of laboratory animals in Europe is accomplished according to recommendations given by the Federation of European Laboratory Animal Science Associations (FELASA). The recommendations list infectious agents and testing intervals for the most frequently used laboratory animal species [9]. Similarly, a joint working group by the AALAS (American Association for Laboratory Animal Science) and the FELASA has published recommendations for a common health reporting format [10]. All previously known rodent-associated viruses appear on the list of the FELASA and of the AALAS-FELASA working group—except MuAstV which has not been included in the list yet. The aim of health surveillance is to monitor the microbiological status of laboratory animal populations as it is well known that the microbiological quality, i.e. undetected infections, of laboratory animals may directly influence experimental variability, and may affect and invalidate research results to a varying degree. Immunodeficient animals are particularly susceptible to viral infections and are most often persistently infected with continuous virus shedding. Infections in immunocompetent animals, however, are most often limited in time, and viremia and excretion is short, with the consequence that virus is often not detected by direct methods. Serological assays instead can use a large window for identifying exposure to infections in the past since antibodies persist for months, Therefore, serological assays are invaluable for health monitoring in detecting antibodies to viruses in laboratory animals.

The mechanisms of astrovirus infection and transmission in mice are largely unknown. The aim of this study was to develop and validate a serological assay that specifically detects antibodies to murine astroviruses and that can be used for health monitoring of mouse colonies. A bead-based multiplex fluorometric antibody binding assay was established in the diagnostic laboratory at the German Cancer Research Center (DKFZ) in Heidelberg, Germany, in cooperation with the Division of Molecular Diagnostics of Oncogenic Infections of the DKFZ and was used in routine diagnostics. Because of the potential to simultaneously detect antibodies against multiple infectious agents in a single reaction, the small sample volume, the high-throughput capability and therewith cost-effectiveness, multiplex serology is advantageous over other serological tests. Therefore, this method is state-of-the-art in mouse colony health monitoring of large diagnostic laboratories. A previously published MuAstV RT-PCR assay was used to define the positive and negative status of DKFZ housing units in order to classify serum samples used to test and validate the serological assay [8]. PCR was also used to confirm MuAstV presence and absence in different units at the DKFZ based on serology results. It can be assumed that with the availability of sensitive and specific tests for the detection of MuAstV and an improved understanding of the mechanisms of infection and transmission interest in this new virus will increase, and that MuAstV will possibly be added to internationally agreed health monitoring standards. The development of this high-throughput assay made the implementation of the first sero-epidemiological study possible demonstrating that MuAstV has a worldwide distribution and that not only immunodeficient but also wildtype and transgenic mice are infected by MuAstV and produce high levels of specific antibodies.

## Materials and methods

### Sourcing and care of mice

The vivarium of the German Cancer Research Center (DKFZ) comprises different units which either have a breeding purpose (access limited to animal care technicians) or a mixed purpose with animal experimentation and breeding in the same unit (with additional access for scientific personnel and research materials). The term unit used in this paper is understood as a self-contained microbiological entity in terms of space, personnel, material and traffic of animals, and follows the definition of the AALAS-FELASA working group [10]. There are six breeding units (barrier unit 1 to 4 with mice housed in open cages, units A and B with individually ventilated cages (IVC)), six experimental units with mixed purpose (barrier unit 5 with open cages, IVC units D, ATV, S2, T137 and Biotechnik), an embryo transfer unit (Labor2000 with isolators) and a quarantine unit (with IVC and isolators). Access to the whole facility is restricted to trained and authorized personnel only. Mixed units can be entered after changing of clothes. Access to the breeding units is only possible via compulsive wet showers.

Throughout the study, the units of the Central Animal Laboratory (CAL; barrier units 1–5, IVC units ATV, T137) were run at about 80% occupancy. In the course of 2015/2016 units A, B, D, S2 and Biotechnik of the new animal facility of the Center for Preclinical Research (CPR) were opened for operation. Animals from the CAL unit ATV were partly moved to S2, animals from T137 were completely moved to unit D and Biotechnik. The central breeding units, units A and B, were opened for operation in February 2016 and March 2015, respectively. All mouse strains were re-derived by embryo transfer before being introduced in units A and B. Mice in units A and B were colonized with a defined flora, the Altered Schaedler Flora, which consists of a cocktail of 8 bacteria species [11]. Defined flora mice were purchased from Taconic Biosciences, USA, and bred in positive pressure isolators. Their offspring was used as foster mothers for re-derivation of strains.

Mice were housed in open cages (1145T, Tecniplast, Buguggiate, Italy), IVC cages (Greenline, Tecniplast, Italy, and Ehret type 1L cages, EHRET Labor- und Pharmatechnik, Emmendingen, Germany) or in flexible film isolators (Metall und Plastik GmbH, Radolfzell, Germany). All cage beddings (aspen material), nesting material (aspen wood, 24–120 mm, Abedd Vertriebs GmbH, Vienna, Austria), food (Mouse and Rat Breeding No. 3307 and Maintenance No. 3437, KLIBA NAFAG, Kaiseraugst, Switzerland) and water were autoclaved before use. Heat sensitive material was introduced after sterilization with H_2_O_2_. Cage changes in barrier units 1–5 were done within the room without changing gloves between cages. Cage changes of IVC were performed under a laminar-flow biosafety cabinet or changing station with glove changes between cages. P3-oxonia active 5% was used for disinfection before and after each cage changing cycle.

The animal facility of the DKFZ has been officially approved by the responsible authority (Regional Council of Karlsruhe, Germany) under the official approval file no. Az 35–9185.64BH DKFZ. Housing conditions are thus in accordance with the German Animal Welfare Act (TierSchG) and the EU Directive 2010/63/EU. Regular inspections of the facility are conducted by the Veterinary Authority of Heidelberg, Germany. Compliance with institutional guidelines and legal regulation regarding care and handling of animals was ensured by designated veterinarians according to article 25 of Directive 2010/63/EU and Animal-Welfare Body according to article 27 of Directive 2010/63/EU.

### Health monitoring

Testing of animals was conducted in the Microbiological Laboratory of the Center for Preclinical Research at the DKFZ. For routine health monitoring colony animals (primarily barrier units 1 to 5, also all other units), bedding sentinels (experimental units with mixed purpose) and contact sentinels (breeding units A and B) were tested periodically for viral and bacterial infections, and parasites. Breeding units 1–4, A, B and also experimental unit 5 were monitored every week with at least 2–4 animals tested. Other experimental units were monitored less often in two to three weeks intervals. In addition diagnostics of sick animals was accomplished whenever necessary. All imported animals were tested upon arrival and kept in quarantine for subsequent re-derivation by embryo transfer. In Labor2000 all recipient females of an isolator were tested to verify the high hygienic status before progeny was transferred into one of the housing units. The MuAstV serological assay was incorporated into the pre-existing multiplex serology and therewith included in routine diagnostics in April 2015. Fecal samples tested for MuAstV RNA were collected during microbiologic monitoring from 2014 until March 2017.

Animal testing in the frame of this study was conducted within health monitoring of laboratory rodent colonies at the DKFZ and was officially approved by the local governmental authorities (Regional Council of Karlsruhe, Germany) under the notification number A-3/02.

### Sera

Altogether 5529 serum samples were tested in the course of this study for MuAstV antibodies: 4556 sera collected in the frame of health monitoring from DKFZ animals, these were also used for assay establishment and validation, 661 serum samples derived from mice imported to the DKFZ from 58 different institutions from 15 countries all over the world and 312 sera of animals from commercials breeders. Blood samples were either collected from live animals by puncture of the tail vein or the Vena temporalis superficialis or during dissection by cardiac puncture after euthanasia with CO_2_. Sera were diluted 1:5 in HA buffer (90 mM NaCl, 3.45 mM NaH_2_PO_4_, 7 mM Na_2_HPO_4_, pH 7.2), kept at 4°C overnight and centrifuged at 2000 × g for 30 minutes. Sera were further diluted 1:50 in pre-incubation buffer (see below) before being incubated with the 31-plex micro-bead mixture. Serological testing for routine health monitoring was run every week.

### RNA preparation and PCR for murine astrovirus detection

Fecal samples were collected from live animals or during necropsy. RNA was extracted from the feces using the Maxwell 16Lev device and the Maxwell 16LEV simplyRNA Tissue Kit (Promega GmbH, Mannheim, Germany). Approximately 10 mg of fecal material was cut from the pellet and added to 200 μl of homogenization solution. The sample was mechanically homogenized by a sample homogenizer (Precellys24, Bertin Instruments, Montigny-le-Bretonneux, France) at 5000 × g for 15 seconds. To the homogenate 200 μl lysis buffer was added before the mixture was transferred into the first well of the Maxwell processing cartridge. For the following steps the manufacturer’s instructions were followed and the RNA was eluted in 50 μl RNAse free water. RNA was transcribed to cDNA using QuantTect Reverse Transcriptase (QIAgen, Hilden, Germany) following the manufacturer’s instructions except that half the reaction volume was used. One microliter template RNA was used for the reaction. In a first step genomic DNA was eliminated with gDNA wipeout buffer followed by a second reverse transcriptase step. Subsequently PCR was done using the Multiplex PCR kit (QIAgen, Hilden, Germany) according to the manufacturer’s instructions except that half the reaction volume was used. Primers MuAstV-BF (5’-GAATTTGACTGGACACGCTTTGA-3’) and MuAstV-BR (5’-GGTTTAACCCACATGCCAAA-3’), targeting a region of 328 base pairs within the RNA-dependent RNA polymerase gene, were previously published by Ng et al. 2013 [8]. One microliter cDNA was added to the reaction mixture. Reaction conditions and the appropriate annealing temperatures for primers (61°C) were adjusted according to the manual supplied by QIAgen. Amplicons were analyzed by ethidium bromide gel electrophoresis.

### Generation of recombinant MuAstV proteins

The amino acid sequences of human astrovirus 1 (UniProt-ID O12792, GenBank accession no S68561.1) and MuAstV (UniProt ID K0C109, corresponds to GenBank accession no JX544743.1, murine astrovirus strain STL 1) were aligned using the program ClustalW by the HUSAR's (Heidelberg Unix Sequence Analysis Resources) web interface W2H to approximate amino acid sequences of homologous capsid protein regions. Resulting amino acid positions (aa) of murine astrovirus specific viral proteins VP70-long, VP70-short, VP34 and VP27 are given in Table 1. Nucleic acid sequences were synthesized by Eurofins Genomics (Ebersberg, Germany) and cloned into pGEX4T3tag between a N-terminal glutathione-S-transferase (GST) and a C-terminal epitope called *tag* derived from the large T-antigen of simian virus 40 [12]. Antigens were expressed as GST-X-*tag* fusion proteins in Escherichia coli BL21 (Novagen-Merck, Darmstadt, Germany). Bacterial lysates were prepared as described elsewhere [12]. Protein concentrations were measured according to Bradford [13]. Full-length protein expression was checked by Coomassie-stained sodium dodecyl sulfate-polyacrylamide gel electrophoresis (SDS-PAGE) and by Western Blot using antibodies against GST and *tag*. A GST capture ELISA was used to estimate concentrations of full-length fusion proteins in the lysates by titration and detection by monoclonal mouse IgG1 anti-*tag* antibody KT3 [12, 14].

**Table 1:** Murine astrovirus recombinant capsid proteins.

Viral protein	Function	Amino acid position^1^	Molecular weight (+ GST in kDa)	Accession number
VP70 long	Premature protein	aa 1–690	61 kDa (86)	UniProt K0C109
VP70 short	Premature protein	aa 1–554	76 kDa (101)	UniProt K0C109
VP34	Capsid shell	aa 1–310	34 kDa (59)	UniProt K0C109
VP27	Spikes	aa 391–681	32 kDa (57)	UniProt K0C109

^1^ position of the capsid protein (ORF2) of MuAstV strain STL1 (GenBank accession no JX544743.1)

### Multiplex MuAstV serology

The multiplex MuAstV serology is based on a GST capture immunosorbent assay combined with the fluorescent bead technology from Luminex Corp. (Austin, Texas, USA). The MuAstV GST-X-*tag* fusion proteins were loaded and affinity-purified directly on individual sets of spectrally distinct glutathione-casein-coupled polystyrene beads that contain embedded fluorescent dyes (SeroMap; Luminex, Austin, TX, USA). General set-up and protocol of the multiplex MuAstV serology is described elsewhere [15, 16]. Briefly, for antigen loading of beads, lysates were diluted in blocking buffer (1 g/l Casein in 1 × PBS, pH 7.4) to give a final concentration of 1 g/l to reach condition of high antigen excess. 10 μl activated beads per plate were added to 1 ml protein lysate. After incubation and washing with blocking buffer beads were resuspended in storage buffer (1 g/l Casein, 0.5 ml/l sodium azide in 1 × PBS). Sera were diluted 1:50 in a serum pre-incubation buffer containing 1 g/l casein, 0.5 g/l polyvinylalcohol, 0.8 g/l polyvinylpyrrolidone and 2 g/l GST-*tag* lysate (total protein lysate from bacteria overexpressing GST-*tag* without adhering MuAstV sequences to block binding of antibodies) in 1 × PBS and incubated to suppress unspecific binding of antibodies to the beads themselves [16]. A bead set coated with anti-mouse immunoglobulin G (AffiniPure Donkey Anti-Mouse IgG; 4 μg/ml beads) was used as an IgG control that allowed differentiation between immunocompetent and immunodeficient animals with reduced amounts of IgG in the serum. Another bead set was coated with bacterial lysates (GST-*tag* lysate) as a negative control. The differently labeled and antigen loaded beads were mixed to be incubated with an equal volume of diluted serum in a single reaction on a 96-well plate. A Luminex analyzer (Lx100 and a BioPlex200, BioRad Laboratories GmbH, Munich, Germany) was used to simultaneously detect the bead set and consequently the bound antigen, and to quantify the amount of bound serum antibody by a secondary antibody (biotinylated goat anti-mouse IgM/IgG, Dianova, Hamburg, Germany; 1:1000 diluted in blocking buffer) and reporter fluorescence (Streptavidin-R-Phycoerythrin, MOSS Inc., Pasadena, Ca, USA; 1:500 diluted in blocking buffer). To assess background reactivity all bead sets were additionally run in parallel without serum. The monoclonal mouse IgG1 anti-*tag* antibody KT3 [14], which recognizes the C-terminal *tag* sequence of the recombinant protein, was used instead of serum and secondary antibody as a full length protein loading control. For each bead set, the quantity of antibodies bound to the respective antigen was reported as the median fluorescence intensity (MFI) of at least 75 beads per antigen per measure. Final antigen-specific MFI values (net values) were calculated by subtracting the individual bead background fluorescence values (negative control, i.e. no serum) and the MFI value of the GST-*tag* (fusion protein domain without antigen). Samples were defined as positive if the net MFI values were above the calculated cut-off.

### Determination of diagnostic sensitivity, specificity, cut-off definition and statistical analyses

Test results of serum samples from 200 supposedly infected and 200 uninfected mice were used to calculate an antigen specific cut-off by a receiver operating characteristic (ROC) curve. The curve was calculated by using PCR results from previous testing as reference standard to calculate the antigen-specific cut-off value from the MFI values. Repeatability of each assay was assessed by running the same internal quality control sera between runs. Sera from routinely monitored and sick animals, respectively, were analyzed weekly for two years, as described in chapter Health monitoring, and yielded similar MFI results (presented in boxplots). Kappa values with their corresponding 95% confidence intervals (CI) were computed to estimate assay concordance (inter-rater agreement) and reproducibility. Statistical analyses and graphical presentation for the overall distributions of net MFI values obtained by multiplex MuAstV serology were performed with GraphPad Prism (GraphPad Software, Inc., La Jolla, CA, USA). Mann-Whitney Rank Sum test was applied to evaluate statistical significance of results yielded in multiplex serology between positive and negative groups using SigmaPlot 13.0 (Systat Software GmbH, Erkrath, Germany).

## Results

### Selection of immunogenic MuAstV proteins

The astrovirus genome contains three open reading frames (ORFs). ORF1a and 1b at the 5’-end of the genome encode the non-structural proteins NSP1a and NSP1ab which contain the protease and the RNA-dependent RNA-polymerase involved in RNA transcription and replication and some other proteins with unknown function. ORF-2 comprises two-thirds of the 3’-terminal genome and encodes the structural protein VP90 which contains a highly conserved N-terminal domain and a C-terminal domain with high sequence variability. The viral particle assembles from VP90 which is translated as the capsid precursor protein [17, 18]. Morphogenesis of infectious particles relies on a cascade of proteolytic cleavages of the capsid precursor protein. In the course of the maturation process VP90 is cleaved by intracellular caspases at the C-terminus to generate VP70 which in turn is required for the release of immature particles from the host cells [17, 19, 20]. After extracellular cleavage by trypsin resulting in three structural proteins VP34, VP27 and VP25, the mature, infectious particle is formed [21]. While VP34, containing the conserved N-terminal domain, builds the capsid shell, the overlapping units VP27 and VP25, containing the variable C-domain, form the dimeric spikes [22, 23].

**Fig 1:**
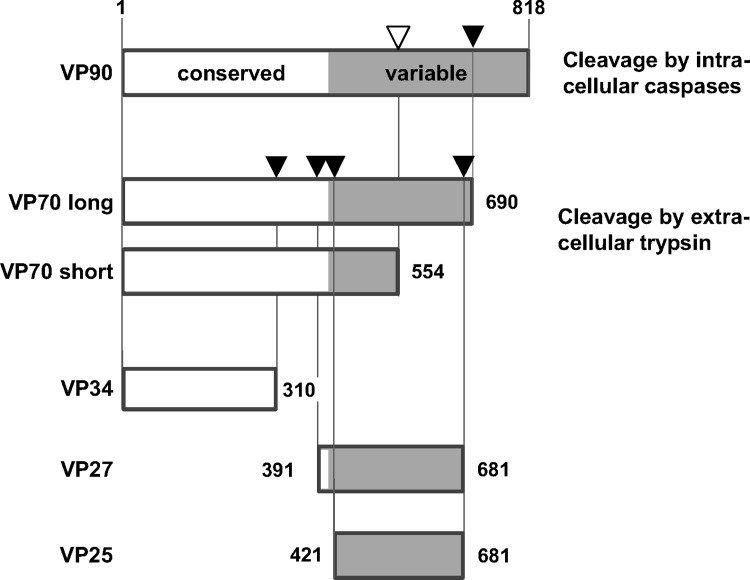
Schematic presentation of capsid processing as basis for the selection of amino acid sequences of MuAstV capsid proteins. The recombinant capsid proteins were used as antigens in multiplex MuAstV serology. VP70 long, VP34 and VP27 sequence fragments are based on processing data of human astrovirus capsid protein according to UniProt ID O12792 [24]. Cleavage sites are indicated by black arrowheads. VP70 short fragment is based on the predicted caspase cleavage site at aa position 554 as suggested by Expasy Peptide Cutter (white arrowhead). The figure was adapted from Krishna et al. [22] and modified by the authors.

In the scope of this study several potentially immunogenic viral proteins were selected on the basis of available data on capsid processing. Since no details on the processing of murine astrovirus particles have been published, sequence sections of MuAstV proteins VP70, VP34 and VP27 were determined by alignment with an equivalent capsid sequence of human astrovirus 1 (HuAstV-1) and in accordance with previously published posttranslational cleavage sites of the capsid protein [24]. Nucleotide sequences of four distinct proteins were synthesized based on a previously published MuAstV sequence (STL1; GenBank accession No. JX544743.1), subcloned and recombinantly expressed as GST-X-*tag* fusion proteins: the premature viral protein VP70 which contains all proteins of the mature viral particle was expressed as VP70 long (aa 1–690; full-length protein after intracellular cleavage of VP90 as described for human astroviruses) and VP70 short (aa 1–554; here the fragment ends at the putative cleavage site amino acid position 554 as being predicted by Expasy Peptide Cutter [25]), the spike protein VP27 and the capsid protein VP34. Fig 1 displays schematically capsid processing and amino acid sequences of the capsid proteins that were expressed to be used as antigens in MuAstV multiplex serology.

### Development of a serological assay for MuAstV antibody detection

**Fig 2:**
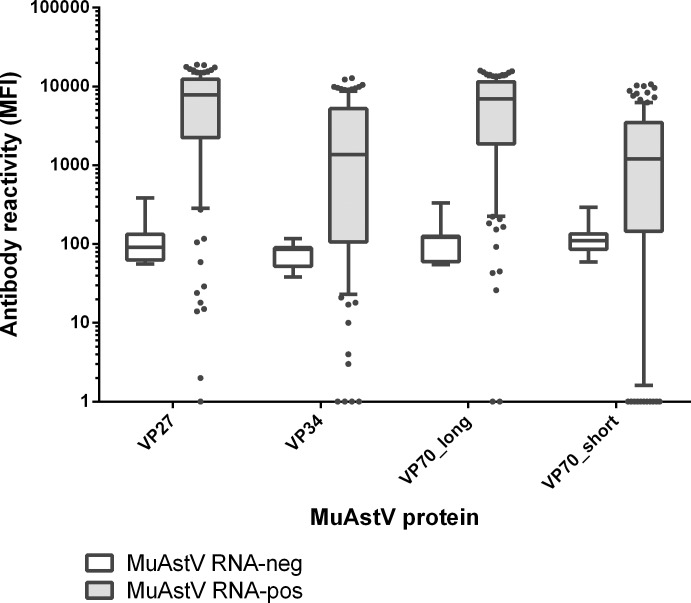
Comparison of antibody reactivities of four MuAstV recombinant proteins in multiplex serology. Box plot of net MFI values (S1 Dataset) are shown. Boxes are delimited by the first and third quartile (IQR, Q1-Q3) and represent 50% of data. Whiskers show the 10^th^ and 90^th^ percentiles, respectively. The dots represent data points beyond the upper and lower 10^th^ percentile. The line inside the boxes represents the median. The distribution of MFI values of the negative sera (n = 7) are shown in white boxes, those of the positive sera (n = 112) in grey boxes. All four antigens show significant differences between positive and negative serum samples (VP27 and VP70 long P < 0.001, VP34 P = 0.008, VP70 short P = 0.015, Mann-Whitney Rank Sum test).

Since attempts to propagate murine astrovirus in vitro have not succeed so far [6], recombinant viral proteins were generated to be used as antigens in a MuAstV specific multiplex serological assay. Proteins were expressed as fusion proteins with a GST-X-*tag* in BL21 *E*. *coli* cells. Affinity purification of the GST-X-tagged proteins was accomplished by direct coupling to glutathione-casein-coupled polystyrene beads. In a first approach the four different antigens, VP70 long, VP70 short, VP34 and VP27, were compared by their reactivities in multiplex fluorescent bead assays with a panel of sera from animals, which had been housed in MuAstV-PCR positive units (n = 112), and from animals with a negative MuAstV status (n = 7). These negative, MuAstV uninfected animals were gnotobiotic mice that had been associated with a defined flora (Altered Schaedler Flora) and were free of any viral infection. As the CPR was building up new breeding units with mice associated with the defined flora in order to achieve a high level hygienic status, only a few animals (n = 7) were available for health monitoring when the four MuAstV antigens were compared by their immunogenicity. MuAstV proteins VP27 and VP70 long showed the highest antibody reactivities in terms of MFI values with median MFI of 7813 and of 6998, respectively, yielding a clear separation between positive and negative samples (P < 0.001, Mann-Whitney Rank sum test, Fig 2).

The background reactivities in the negative serum panel of both antigens, VP27 and VP70 long, were similarly low with a median MFI of 91 and 122, respectively. High reactivities against VP34 and VP70 short were also frequent but MFI values were generally lower (median MFI of 1369 and 1203) and MFI differences between the positive and the negative groups were not as pronounced as seen in VP70 long and VP27 (P = 0.008 and P = 0.015, Mann-Whitney Rank Sum test). For the subsequent sero-epidemiological study VP27 was chosen as it showed a slightly higher reactivity and lower background reactivity. The VP27-coated beads were incorporated in the already existing multiplex serology to be used in routine diagnostics of mice.

### Diagnostic sensitivity, specificity, cut-off determination and assay validation

**Fig 3:**
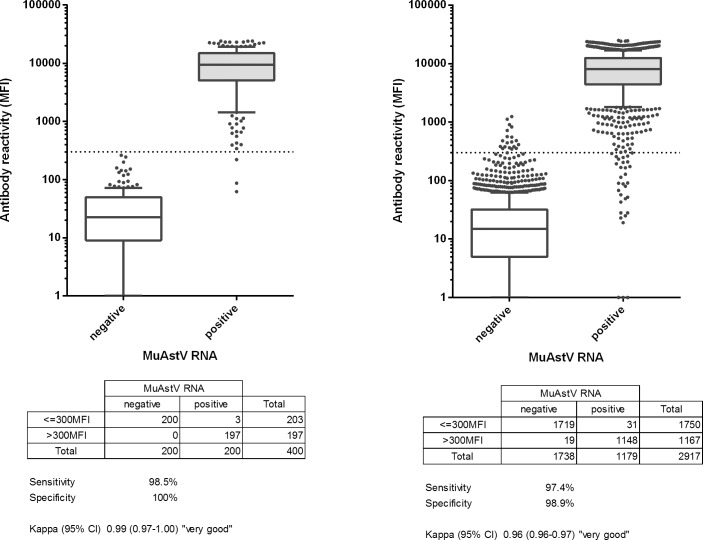
Box plots of net median fluorescence intensity values of test and validation set obtained by multiplex MuAstV serology. The line inside the boxes represents the median, the boxes are delimited by the first and third quartile, and whiskers show the 10^th^ and 90^th^ percentile. The horizontal dotted line indicates the antigen specific cut-off value that gives the optimal diagnostic sensitivity and specificity. The MuAstV capsid protein VP27 in the test and validation set showed highly significant differences of positive versus negative sera (P < 0.001, Mann-Whitney Rank Sum test). Receiver operator characteristic (ROC) analysis and determination of diagnostic sensitivity and specificity were calculated using serum samples from an infected and an uninfected population defined by PCR results. In each panel, the white box plots on the left represent the negative sample set and the grey box plots on the right the positive sample set. (A) Test set (n = 400), (B) Validation set (n = 2917) (S2 Dataset).

To accurately assess the diagnostic sensitivity and specificity of the MuAstV serological assay, samples from units with a known MuAstV PCR status were used. MuAstV serology was compared with a previously published MuAstV PCR due to a lack of alternative serological testing methods [8]. For assay testing, a panel of 400 serum samples was used. A set of 200 sera of animals from DKFZ barrier units 1 to 5, with a known positive MuAstV PCR status and presumably high numbers of infected animals due to the open caging system, were classified as positive sera. The MuAstV negative serum set included 200 samples derived from astrovirus-free mice (gnotobiotic animals associated with the Altered Schaedler Flora and germfree mice) from breeding units A, B and Labor2000 with no history of any viral infection. ROC analysis showed an estimated sensitivity and specificity of 98.5% and 100%, respectively, at a suggested cut-off of 300 MFI. At this cut-off, 197 assumed infections were concordantly positive, 3 sera were discordantly negative and 200 serum samples of animals from MuAstV negative units were consistently negative, yielding an overall kappa of 0.99 (CI.95 = 0.97–1.00) and thus showing an excellent agreement with PCR results. Box plots comprising the MFI data of the test set are presented in Fig 3A. Values of the concordantly MuAstV positive samples of the positive test set ranged from 341 to 24019 MFI with a median MFI value of 9390, whereas the negative test set showed a median MFI of 22. The results showed that MuAstV specific antibodies were detected with high sensitivity and specificity by the newly developed serological assay. As MFI values were measured in multiple runs on different days the analysis also showed a good inter-assay repeatability.

Based on the sensitivity and specificity calculation made to plot the ROC curve from the 400 mouse sera, a MFI value of 300 was used as a cut-off to discriminate negative and positive test results in all subsequent serum samples tested in this study. In the following study, the calculated test characteristics were validated with a total of 2917 serum samples comprising a set of 1738 negative sera from animals from breeding units A, B and the embryo transfer unit Labor2000, units with a negative MuAstV status, and a set of 1179 sera from animals which had been housed in barrier units 1 to 5, units which were defined MuAstV positive by PCR. Test sensitivity and specificity resulted in comparable results of 97.4% and 98.9%, respectively. The overall kappa value yielded 0.96 (CI.95 = 0.96–0.97) and was thus assessed as very good demonstrating a high level of testing agreement among PCR and serology results. A median MFI value of 8053 was calculated for the positive validation set, a value of 14.5 for the negative validation set. Assay robustness was evaluated by repeated, weekly measurements over a time period of 24 months with overall similar MFI values shown by the box plots in Fig 3B.

### Prevalence of MuAstV in research mice at DKFZ and spread within different housing systems

After comparison of antigenicity of recombinant astrovirus capsid proteins, the MuAstV capsid protein VP27 coupled bead set was integrated into the preexisting multiplex serology that was used to test antibodies specific to murine-associated viruses and bacteria. After cut-off calculation as described above the value was then applied to all samples tested in the course of health monitoring to decide on the sero-status. From April 2015 until March 2017 a total number of 4556 sera from mice at the DKFZ, including the test and validation set described in the previous section, were analyzed by multiplex serology for IgM and IgG antibodies for evidence of a previous MuAstV infection (S3 Dataset). About 50% of serum samples tested in the MuAstV assay were derived from genetically modified mice. Of the wildtype mice, CD1 was the most frequently tested strain (38%) due to testing of high numbers of recipient females used in embryo transfer. Wildtype strains C57BL/6, BALB/c, NMRI and FVB/N were monitored to a lesser extent with 7.5%, 1.8%, 0.96% and 0.9%, respectively. The overall prevalence of MuAstV antibodies in serum samples was 38% (n = 1739). Total numbers of mice tested per unit, minimum, maximum and median MFI values are summarized in Table 2.

**Table 2:** Comparison of MuAstV antibody prevalence, antibody levels and testing characteristics between breeding and experimental units at the DKFZ.

		MuAstV sero-prevalence at the DKFZ		Test characteristics			
Purpose	Unit	No. of tested	No. of positive (percentage)	Min (MFI)	Max (MFI)	Median (MFI)	IQR* (25%-75%)
B	Units A/B	866	14 (1.6%)	1	1 241	13	4–35
B	Labor2000	1072	5 (0.5%)	1	721	16	6–33
B	Units 1–4	890	866 (97%)	19	24 874	7604	3 792–12 989
E	Unit 5	489	479 (98%)	1	23 573	9071	5 797–12 569
E	ATV	518	165 (32%)	1	20 847	38	8–1 190
E	Biotechnik	110	9 (8%)	1	14 524	29	11–77
E	Unit D	153	74 (48%)	1	23 061	164	17–7 721
E	S2	42	28 (67%)	1	12 836	2979	101–8 082
E	T137	416	99 (24%)	1	20 658	21	8–200
Sum		4556	1739 (38%)				

*IQR (Q1; Q3) refers to the first and third quartiles, respectively, of the sampling-adjusted distribution of antibody levels (MFI values)

(B) breeding unit, (E) experimental unit with breeding

**Fig 4:**
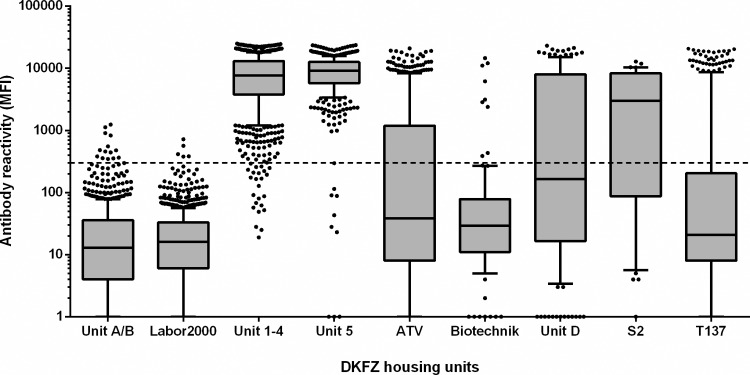
Comparison of murine astrovirus specific antibody detection in DKFZ breeding and experimental units by multiplex serology. Box plot of net MFI values (S3 Dataset) are shown. Boxes are delimited by the first and third quartile (IQR, Q1-Q3) and represent 50% of data. Whiskers show the 10^th^ and 90^th^ percentiles, respectively. The line inside the boxes represents the median. The horizontal dotted line indicates the antigen-specific cut-off value. The distribution of antibody reactivities in terms of MFI values ranges from units without virus circulation (units A/B, Labor2000), units with a low to medium sero-prevalence (ATV, S2, T137, Biotechnik and unit D) to units where most animals had been in contact with the virus (units 1–4 and 5).

In CAL barrier units 1 to 5 MuAstV antibodies were present in 97.5% (n = 1345) of mice. This was attributed to housing in open cages which facilitates the spread of infections from cage to cage. As expected the new CPR breeding units A and B, which had been built up with animals colonized with a defined flora and in which mice could only be introduced by embryo transfer, very few samples (14 of 866) showed reactions in MuAstV serology with MFI values slightly above the cut-off which was attributed to non-specific antibody reactivities. In experimental units with individually ventilated cages (ATV, S2, T137, Biotechnik and unit D) sero-prevalence ranged from 8% to 67% (n = 375). Prevalence was highly dependent on the hygienic level of the experimental unit, the introduction of animals from commercial breeders and on the number of scientific groups working within the unit. The Biotechnik unit, for instance, housed mice with a very high hygienic status and introduction of animals from commercial breeders was rare, opposite to the other experimental units, ATV, S2, T137 and unit D. Fig 4 shows the distribution of MuAstV reactivities in terms of MFI values comparing the breeding units (units A/B, Labor2000, units 1–4) and the experimental units with a low prevalence of murine astrovirus antibodies (Biotechnik, T137), medium prevalence (ATV, unit D, S2) and unit 5 with nearly 100% of animals with a previous MuAstV infection.

PCR testing of the different units was performed before MuAstV serology was available and was intensified during this study to verify multiplex serology results. As PCR testing is time-consuming and labor-intensive, and only a limited number of samples can be processed at the same time, sample numbers are much lower compared to serology data. Table 3 displays results of the PCR analysis of mice from the different DKFZ units summed up in four age groups. Virus prevalence ranged from 0% to 76%. Interestingly, infection and therewith excretion with MuAstV was not age related, even in units with open cages, and most likely independent of the presence of specific antibodies as most animals tested were sero-positive. Fecal samples from immunodeficient mice (i. a. NOD scid gamma, Rag2 knockout, Athymic Nude-Foxn1^nu^, BALB/c Nude, in square brackets) were mostly tested positive by PCR.

**Table 3:** Prevalence of MuAstV RNA in mice at the DKFZ versus age of mice and housing unit.

Age of mice		5–10 weeks		11–20 weeks		21–30 weeks		> 30 weeks		Sum	
Unit	cage type	positive/total no. of mice tested	percent positive	positive/total no. of mice tested	percent positive	positive/total no. of mice tested	percent positive	positive/total no. of mice tested	percent positive	positive/sum mice tested	percent positive
Unit 1	open	1/5	20%	8/17 [8/8]	47%	1/9 [1/1]	11%	2/9 [1/1]	22%	12/40	30%
Unit 2	open	4/9	44%	10/19	53%	2/6	33%	1/2	50%	17/36	47%
Unit 3	open	7/8	88%	10/10	100%	4/10	40%	4/6	67%	25/34	74%
Unit 4	open	7/10 [1/1]	70%	11/14 [4/4]	79%	6/8 [2/2]	75%	4/5	80%	28/37	76%
Unit 5	open	9/14	64%	19/30	63%	9/17 [2/2]	53%	5/11	45%	42/72	58%
Unit A	IVC	0/5	0%	0/7	0%	0/7	0%	0/9	0%	0/28	0%
Unit B	IVC	0/43 [0/30]	0%	0/20 [0/2]	0%	0/9	0%	0/9 [0/4]	0%	0/81	0%
Unit D	IVC	4/5 [1/1]	80%	5/10 [2/2]	50%	10/16 [6/6]	63%	1/3	33%	20/34	59%
ATV	IVC	4/11	36%	5/17 [3/3]	29%	2/14 [1/1]	14%	4/16 [1/1]	25%	15/58	26%
Biotechnik	IVC	1/7 [0/1]	14%	0/6	0%	2/14	14%	1/14	7%	4/41	10%
T137	IVC	0/1	0%	2/8	25%	2/4	50%	2/4 [1/1]	50%	6/17	35%
S2	IVC	4/10	40%	3/7	43%	7/8 [7/7]	88%	2/7 [0/1]	29%	16/32	50%
Labor2000 (DF)	Isolator	0/14	0%	0/14	0%	0/11	0%			0/39	0%
	Sum	41/142	29%	73/179	41%	45/133	34%	26/95	27%	185/549	**34%**

[ID] number of immune-deficient mice (included in total number); DF = defined flora (gnotobiotic mice)

### Antibody and virus prevalence in mice from commercial breeders

To obtain an estimate of the sero-prevalence of murine astrovirus in mice purchased from commercial breeders, from April 2015 until March 2017 a total number of 312 mice from six commercial breeders were screened for specific antibodies in order to check the hygienic status. The number of mice tested per commercial breeder and the selection of strains roughly followed the acquisition of mice by researchers at the DKFZ. Primarily wildtype strains were purchased, i.e. FVB/N, C3H/HeN, BALB/c, NMRI, C57BL/6J, C57BL/6N and CD1, but genetically modified and immunodeficient mice were also tested. All breeders operate breeding units in Europe and/or USA. One breeder sent animals from three locations (different countries), described here as A1, A2 and A3, and thus the three locations were assessed separately. Mice from all six commercial breeders were tested positive by serology, though with regard to breeder C only one serum out of 18 showed reactivity with MuAstV VP27 which may therefore be considered as false positive. MuAstV specific antibodies were detected in 100% (n = 86), 100% (n = 8), 5.6% (n = 1), 56% (n = 105), 66% (n = 4) and 100% (n = 4) of mice from breeder A1, B, C, D, E and F whereas breeder A2 (n = 5) was tested negative (Table 4, S4 Dataset). In addition, fecal samples were checked by PCR for the presence of MuAstV genome and resulted in a variable picture of virus excretion, with 0% to 100% of mice positive for virus RNA (Table 4). MuAstV findings in animals from commercial breeders had been reported earlier [8], though screening was not done on a broad scale.

**Table 4:** MuAstV antibody and virus (RNA) prevalence in mice from commercial breeders.

	Serology			PCR	
Breeder	No. animals tested	Positivity (percentage)	Median MFI (IQR)	No. animals tested [ID]	Positivity (percentage)
A1	86	86 (100%)	12.176 (Q1-Q3; 7.061–17.161)	23 [1]	23 [1] (100%)
A2 (serology, PCR), A3 (PCR)	5	0 (0%)	144 (Q1-Q3; 121–147)	7 [3]	4 [3] (57%)
B	8	8 (100%)	18.100 (Q1-Q3; 10.810–20.255)	3	3 (100%)
C	18	1 (5.6%)	23 (Q1-Q3; 2–46)	6	0 (0%)
D	185	105 (56%)	2.734 (Q1-Q3; 46–11442)	18	8 (44%)
E	6	4 (66%)	6.247 (Q1-Q3; 1.267–7.463)	10	6 (60%)
F	4	4 (100%)	9.840 (Q1-Q3; 6.539–13.938)	7	7 (100%)

(IQR) Interquartile range (Q1-Q3); [ID] number of immune-deficient mice (included in total number)

### Worldwide distribution of MuAstV in laboratory mice research colonies

**Fig 5:**
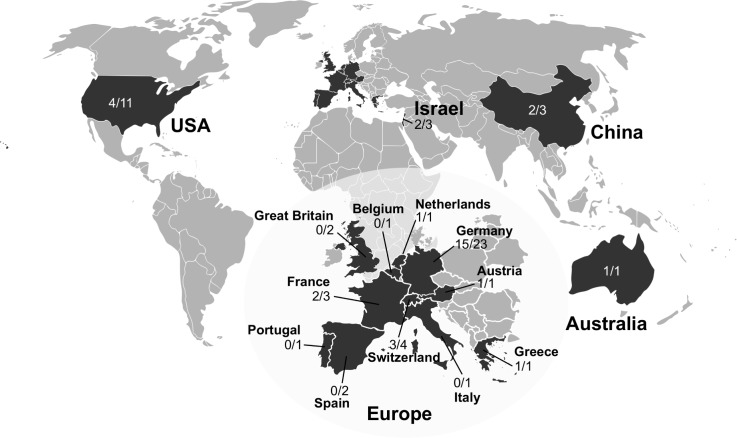
MuAstV positive institutes worldwide based on serology data of imported animals to the DKFZ from 2015 until March 2017. Mice from a total of 58 institutes in 15 different countries were tested by multiplex MuAstV serology (S5 Dataset). The number of research institutes that sent sero-positive mice divided by the total number of institutes tested is presented for each country. Creative Commons BlankMap-World gray by Vardion 2006 (https://commons.wikimedia.org/wiki/File:BlankMap-World_gray.svg) licensed under CC BY-SA 3.0 was used as map basis and modified by the authors.

With the importance of scientific collaborations, the increased availability of transgenic mice and their use in biomedical research the global exchange of transgenic mice has increased significantly in recent years. Therefore importations of mice from other research institutions have become daily business for laboratory animal facilities. As imported animals always pose a risk to the hygienic status of mouse colonies by unintentional introduction of infectious agents, they are usually quarantined and microbiologically tested before transferred into a breeding or experimental unit. Mice imported to the DKFZ were housed in quarantine and tested upon arrival to evaluate the risk of pathogen introduction. For over two years, starting in 2015 until March 2017, 661 mouse sera from imported animals were screened by multiplex serology for the presence of antibodies against rodent-specific viruses and bacteria, including murine astroviruses. In addition, 203 mice were also tested for MuAstV shedding in feces by PCR (S5 Dataset). Transgenic strains were obtained from 58 research institutions from 15 different countries all over the world (America, Asia, Australia and Europe), with Germany the most frequent source. Table 5 lists the countries of mouse origin, the number of scientific institutes and the total number of mice tested by serology and by PCR. Of 661 serologically tested mice, 253 mice (38%) showed antibodies against MuAstV. The percentage of institutes that sent seropositive animals was relatively high with 55% (n = 32). Serology results correlated well with MuAstV RNA detection, though the number of institutes that sent animals excreting MuAstV (RNA positive) was slightly lower with 46% (n = 11). Again data acquired by PCR testing were less in comparison to the amount of data yielded by serology since PCR testing was relatively laborious and time-consuming. The worldwide distribution of scientific institutes from which MuAstV positive mice were imported to the DKFZ is depicted in Fig 5 and demonstrates that MuAstV infections are widespread in contemporary laboratory mouse colonies nowadays.

**Table 5:** Multiplex MuAstV serology and PCR testing of mice imported to the DKFZ, Germany, from 2015 until March 2017.

	Method	Multiplex Serology^a^				PCR^b^			
	Country	No. of institutes tested	Total no. of mice tested	No. of positive mice	No. of positive institutes	No. of institutes tested	Total no. of mice tested [ID]	No. of positive mice [ID]	No. of positive institutes
America	USA	11	127	33	4	5	44	8	2
Asia	China	3	22	9	2				
	Israel	3	18	9	2				
Australia	Australia	1	15	12	1	1	4	4	1
Europe	Austria	1	6	4	1				
	Belgium	1	4	0	0				
	France^1^	3	20	7	2	2	12	4	1
	Germany^1^	23	319	146	15	11	89 [23]	27 [21]	4
	Great Britain	2	45	0	0	1	15	0	0
	Greece	1	13	13	1	1	13	4	1
	Italy^1^	1	10	0	0				
	Netherlands	1	10	10	1	1	9	9	1
	Portugal	1	5	0	0	1	5	0	0
	Spain^1^	2	16	0	0				
	Switzerland	4	31	10	3	1	12 [5]	8 [3]	1
Sum		58	661	253 (38%)	32 (55%)	24	203 [28]	64 (32%)	11 (46%)

Countries from which animals originate are given in the second column.

(a) only immunocompetent mice were counted

(b) immunocompetent and immunodeficient mice were counted; [ID] number of immune-deficient mice (included in total number)

^1^ institutes with 1–2 mice with MFI values slightly above the cut-off were counted as negative when no other animals were unequivocally positive.

## Discussion

This paper describes the development and validation of the first serological assay for the detection of astrovirus infections in laboratory mice. The multiplex MuAstV serology assay based on the VP27 capsid protein was then used in routine health monitoring of rodents at the DKFZ.

A microsphere-based multiplex fluorescent antibody binding assay was chosen since it combines several benefits for its use in rodent health monitoring. Due to its ability to multiplex and monitor antibodies against multiple pathogens simultaneously in a single sample (31 different antigens in this study), the high sensitivity, high sample through-put and small sample volumes required makes its application in serological diagnosis of rodent-specific viral infections highly advantageous [26]. Although this is the first description of a serological assay for the diagnosis of astrovirus infections in mice other assay formats such as ELISA and indirect immunofluorescence had been developed earlier for the diagnosis of astroviruses in chicken [27, 28], ducks [29] and in humans [30].

Due to the lack of knowledge about the structural and immunogenic functions of the structural proteins of MuAstV, four different proteins of the ORF-2 were expressed as GST-X-*tag* fusion proteins in E. coli, i. e. VP70 long, VP70 short, VP34 and VP27, and compared by their reactivities in the bead-based multiplex serology. As a result, antibody reactivity against VP27 und VP70 long capsid proteins showed best reactions and demonstrated highly significant MFI differences between positive and negative mice. VP27 was selected for further assay validation and the following sero-epidemiological study since the spike protein is considered to be the main antigenic protein of the outer particle surface [31–34]. A ROC curve was generated comparing the MuAstV MFI values to PCR results. Accuracy of the diagnostic test was very high with 0.99 resulting in 98.5% sensitivity and 100% specificity at a cut-off value of 300 MFI. The assay was validated using a large number of serum samples from mice with a predicted reactivity on the basis of PCR results and yielded similar testing characteristics with 97.4% sensitivity and 98.9% specificity. In addition, the assay demonstrated a high repeatability and robustness since multiple serum samples from every week testing were used for assay validation and yielded similar results. Although concordance with PCR results was high, a few animals were discordantly classified by multiplex serology as negative which resulted in the lower sensitivity in comparison to the test specificity. This observation was attributed to animals with no history of astrovirus infection though having been housed in virus-positive units. These mice were defined by serology to be MuAstV antibody-free. The result was not surprising, since even in units with open caging system, where a widespread distribution of infections is assumed, not all animals may have been infected by MuAstV or are possibly in the active phase of infection before antibodies are elicited. In particular young animals or animals from units with a negative MuAstV status that have been introduced only recently into units where MuAstV is circulating may not have been in contact with the virus for long enough to make an antibody response.

In this study, the multiplex serological assay was used to evaluate the presence of MuAstV antibodies in laboratory mice housed at the DKFZ animal facility. Of the 4556 samples collected and tested by multiplex serology 38% were tested positive for antibodies. MuAstV sero-prevalence of the different DKFZ units differed strongly and correlated with the cage type. Antibody prevalence varied from high prevalences in units with open cages to lower prevalences in experimental units, in which mice were housed in individually ventilated cages, and was nearly absent in units with housing of gnotobiotic mice. Overall, testing of a large number of mouse sera revealed that MuAstV is widespread within DKFZ units and is in fact the most prevalent infectious agent found. In order to confirm these results mice from different units were tested by PCR for astrovirus shedding in feces. Altogether, MuAstV RNA detection correlated well with serology results. An aspect that was addressed by PCR analysis was the age of mice tested. Under natural conditions, animals are usually infected at a young age. The infection elicits the cellular and antibody response which usually results in virus elimination. The specific immune response prevents or delimits re-infection and shedding. It is conceivable, therefore, that natural MuAstV infection primarily occurs in mice at a young age followed by virus excretion in the feces, and that those mice having experienced an astrovirus infection exhibit specific antibodies and are protected against reinfection. Successful experimental infection of mice at different ages, pups and adult mice between 3 and 12 weeks of age, has been reported in the literature [35–37]. Interestingly, even in units with open cages and high numbers of sero-positive animals MuAstV infection and excretion seemed independent on the age of mice since no age-specific prevalence of MuAstV was observed. These findings strongly suggest that antibodies do not provide protection against re-infection and shedding, and that mice can be infected by MuAstV several times in their life. An earlier study by Yokoyama et al. addressed the role of the innate and adaptive immune response in mice and concluded that B and T cells are required to control astrovirus replication and shedding in feces, but repeated infections in individual animals had not been examined [37]. Another study highlighted the importance of type I interferons in restricting virus replication and controlling MuAstV infection [36]. The authors found that the IFNaR deficient mice used in their study were persistently infected with MuAstV. It was, therefore, impossible to determine whether the immune response was unable to clear the infection or persistent infection was due to reinfection. Persistent MuAstV infection and shedding in immunocompromised Rag1 deficient and IFNaR deficient mice was also reported by others [36, 37]. Similarly high numbers of PCR positive immunodeficient mice in this study indicate that, depending on the type and extend of immunodeficiency, immunocompromised mice show long-term excretion and thus may pose a great risk for virus transmission to naïve, so far uninfected animals. The ability of the virus to re-infect mice with a functional immune system may be a consequence of the high variability of the capsid protein due to the high mutation rate seen in RNA viruses. This may lead to high numbers of virus variants circulating within a population resulting in immune-evasion due to modified antibody binding sites on the virus surface. It is also conceivable that in immunocompetent mice the infection is limited to the intestinal tract, as observed in wildtype C57BL/6J mice by Yokoyama et al. [37], so that the systemic antibody response has no effect on virus shedding. In humans there is evidence that neutralizing antibodies produced in the course of an infection provide protection against re-infection and disease [38, 39]. Since disease symptoms are almost exclusively restricted to young, elderly, and immune-compromised people, it is assumed that anti-astrovirus antibodies protect healthy adults from re-infection [40–42]. In contrary, experiments with turkey astrovirus demonstrated that antibodies are not essential in controlling astrovirus infection [43].

In the frame of health surveillance, mice imported to the DKFZ between 2015 and 2017 were microbiologically tested and also screened for MuAstV RNA and antibodies. 661 serum samples of mice from 15 different countries were tested by serology and 38% were positive for MuAstV antibodies. Virus prevalence (RNA) was comparable with 32% indicating widespread distribution of MuAstV in research colonies worldwide. A widespread occurrence of MuAstV infections in laboratory mice within Japan and the USA has previously been shown by Ng et al. [8]. Against the background of its recent (re-)discovery, due to the absence of clinical symptoms in the course of an infection and the lack of suitable diagnostic tests, high prevalences are not surprising. Similarly, the serology assay was used to screen mice purchased from commercial breeders within Europe and the USA. MuAstV was present in mouse colonies of at least five out of six commercial breeders. These findings coincide with two other studies where MuAstV was found in mice from commercial vendors in the USA [8, 37]. Prevalent astrovirus infections in commercial breeders may have implications for research institutions in pursuing virus-free colonies.

At present the pathogenesis of astrovirus infections in mice is not clear. In the course of this study no pathological findings or symptoms which may be related to an astrovirus infection were observed in any of the wildtype, immunocompetent or immunodeficient transgenic mice. Also infection studies with CD1 pups and adult mice by Compton et al. showed the absence of clinical signs and histo-pathological changes [35]. In other host species astroviruses can cause various disease patterns: in ducks infections cause duck viral hepatitis, a highly contagious and fatal disease in young animals [44–46], and in poultry virus infections result in enteritis, growth defects and mortality [47–49]. Since in this study wildtype and a great number of diverse genetically modified mice were analyzed for virus and antibody prevalence and no differences in prevalences were noticed, it can be assumed that different mouse strains, regardless of immune-competence, are equally susceptible to astrovirus infection. The higher virus prevalence in immunodeficient mice reported in the literature [8] is rather due to virus persistence in mouse strains with compromised immune systems. Although MuAstV does not cause disease in mice, subclinical infections may alter physiological parameters and immune phenotypes. As a consequence, differences in the infection status between facilities or units, or even between groups of experimental and control animals, may contribute to altered experimental outcomes. Currently, it is not known what effects MuAstV has on the immune system and what impact these effects may have on research. Generally, in order to interpret data obtained from experiments based on small-animal models of disease pathogenesis an accurate recording of factors that may interfere with the immune response is required. Moreover, there is increasing evidence that the microbiota influences disease development in both humans and animals, and that, therefore, the microbiota of animal models may have an essential impact on experimental outcome [50–53]. Health monitoring accounts all known virus infections in research animals, for which testing methods are available [9]. MuAstV diagnostic tests will become important if evidence is found that the virus affects physiological parameters and has, therefore, the potential to invalidate results from animal experiments. In addition, it can be expected that, as soon as reliable diagnostic tools are available, MuAstV will be included in health monitoring programs of scientific institutions aiming at the exclusion of the virus from laboratory rodent populations.

The objective of this work was the development of a sensitive and specific serological assay with high-throughput capability for MuAstV diagnostics. This assay provides the necessary prerequisite to define the MuAstV infection status of laboratory mouse populations which in turn enables the establishment of MuAstV-free mouse colonies. In conclusion, the serological MuAstV assay in this study constitutes a sensitive, specific and robust assay that allows detecting individuals with current or past MuAstV infections. Due to its multiplex format it is convenient for the detection of antibodies to different antigens simultaneously and for high-throughput testing and is, therefore, well suited to be used in health monitoring of rodent colonies. Similarly, it provides a useful and cost-effective tool for large epidemiological studies assessing MuAstV status. The strength of the epidemiologic part of our study was a large sample size with a total of 5529 serum sample tested. Our data demonstrate that MuAstV is highly infectious with prevalences of up to 100% in units with open caging systems. High prevalence rates in mice from our institute and from other research institutes demonstrate that MuAstV infections are widespread in contemporary laboratory mouse facilities. The results indicate that MuAstV is presumably one of the most prevalent if not the most prevalent infectious agent in laboratory mouse colonies worldwide. With the provision of a new diagnostic tool to assess MuAstV infections this study has advanced our understanding of astrovirus transmission in research mouse colonies.

## Supporting information

S1 Dataset Spreadsheet with net MFI values for comparison of recombinant MuAstV proteins. These data were used to generate Fig 2.
(XLSX)

S2 Dataset Spreadsheet with net MFI values of the test and validation set. These data were used to generate Fig 3.
(XLSX)

S3 Dataset Spreadsheet with net MFI values for comparison of DKFZ units. These data are found in Table 2 and were used to generate Fig 4.
(XLSX)

S4 Dataset Spreadsheet with net MFI values of mice from commercial breeders. These data are found in Table 4.
(XLSX)

S5 Dataset Spreadsheet with net MFI values and PCR results of imported mice to the DKFZ. These data are found in Table 5 and were used to generate Fig 5.
(XLSX)
